# Unilateral internal carotid artery agenesis: Correct diagnosis using computed tomography (CT) scan

**Published:** 2018-04-04

**Authors:** Mohammad Reza Sasani, Ali Reza Dehghan

**Affiliations:** 1 Medical Imaging Research Center, Shiraz University of Medical Sciences, Shiraz, Iran; 2 Department of Radiology, School of Medicine, Shiraz University of Medical Sciences, Shiraz, Iran

**Keywords:** Carotid Artery Diseases, Congenital Abnormalities, Tomography, Spiral Computed, Ultrasound Imaging, Doppler Ultrasonography

A 57-year-old woman referred to our radiology department to perform computed tomography (CT) scan because of the left ear pain and suspicious for mastoiditis. Contrast enhanced CT scan of skull base (Figure 1) incidentally revealed the absence of left carotid canal and left internal carotid artery (ICA); the findings were in favor of the left ICA agenesis.

In color Doppler ultrasonography, evaluation of the common carotid artery (CCA), ICA, and external carotid artery (ECA) in right side showed normal flow pattern. Caliber of the left CCA was smaller than right side; there was no evidence of left CCA bifurcation. Left ICA was not visualized, and left ECA was in continuity with ipsilateral CCA. In Doppler waveform, left CCA had little flow in diastole (high resistant flow), similar to left ECA.

Agenesis of the ICA is a rare anomaly. Noninvasive methods such as ultrasound and CT scan are useful in correct diagnosis. In the literature, different flow patterns are described for the ipsilateral CCA and ECA. Yilmaz, et al.^1^ presented a case of congenital absence of the left ICA with normal flow of the CCA, and low resistance flow pattern of the ECA. Dinc, et al.^2^ reported ICA agenesis cases with high resistance flow pattern of the ipsilateral CCA and ECA. 

Various flow patterns may be attributed to the type of collateral circulation associated with the ICA agenesis. In this condition, various collateral pathways are explained that three main types are collateralization through the circle of the Willis (most common), via persistent fetal circulation, and from the ECA.^3^ In case of collateralization from the ECA, CCA could have normal Doppler waveform with low resistant flow of the ECA.^1^ If there is collateral flow through the circle of the Willis, the CCA and ECA could have high resistant flow.

In our case, spectral waveform in left CCA was similar to high resistant flow of the ECA. Therefore, it seems that blood perfusion of left cerebral hemisphere is supplied from collateral pathway through the circle of Willis. Unfortunately, the patient was lost to our follow-up, and further work up was not possible.

**Figure 1:**
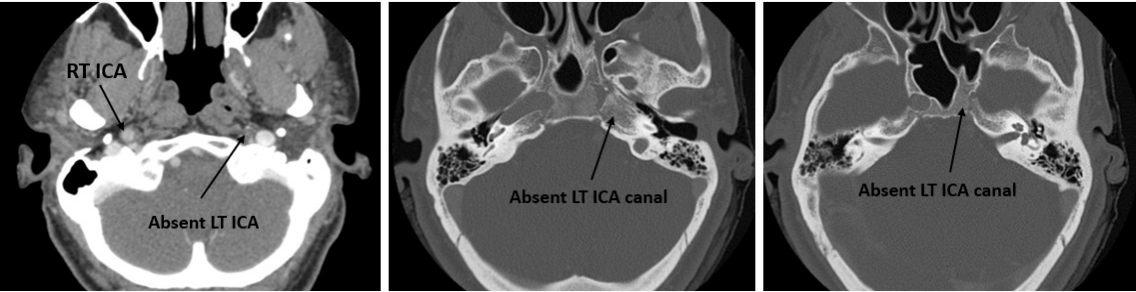
Computed tomography (CT) scan images of the skull base; the absence of left internal carotid artery (ICA) and corresponding bony canal is visible

In cases of ICA agenesis, there are different flow patterns of the ipsilateral CCA and ECA. Despite these variations, correct diagnosis of the ICA agenesis is possible with noninvasive modalities including ultrasound and skull base CT scan.

## Notes:

**How to cite this article:** Sasani MR, Dehghan AR. Unilateral internal carotid artery agenesis: Correct diagnosis using computed tomography (CT) scan. Iran J Neurol 2018; 17(2): 97-8.
